# Commentary: An interactive videogame for arm and hand exercise in people with Parkinson's disease: A randomized controlled trial

**DOI:** 10.3389/fnins.2018.00328

**Published:** 2018-05-15

**Authors:** Veeral Desai, Arnav Gupta, Michael Wong

**Affiliations:** Bachelor of Health Sciences Program, McMaster University, Hamilton, ON, Canada

**Keywords:** exergames, Parkinson's disease, rehabilitation, forced exercise, fine motor movement, gross motor movement

## Introduction

Parkinson's disease (PD) is a neurodegenerative disorder that primarily impairs the motor system, affecting one in every 100 people over the age of 60 (Tysnes and Storstein, 2017). As such, movement-based video games (exergames) have become increasingly popular as a rehabilitation method (Tieri et al., 2018). A study by Allen et al. (2017) examined the potential benefits of a custom-made exergame in the rehabilitation of upper extremity movement among patients with PD, as measured with the nine-hole peg test (9HPT). The study found that after a 12-week period, the use of the exergame did not translate to improvement on the 9HPT nor the majority of the secondary outcome measures (e.g., *hand reaction time*). In this commentary, we attribute the lack of observed improvement to the sedentary nature of the exergame; and suggest more physically demanding exercises are required for motor function improvement in patients with PD, as shown by several studies in the literature.

## Exercise intensity

The 9HPT, a test of manual dexterity, has been shown to be clinically useful in assessing the upper extremity function of patients with PD (Earhart et al., 2011). The Allen et al. (2017) study did not find that patients with PD improved on the 9HPT after they had played a custom-made exergame. A potential explanation for this result is the patients in Allen et al. (2017) did not have upper limb deficits. Alternatively, perhaps the patients in the study did not engage in a physically demanding exergame, playing an exergame that required minimal intensity, with movements that focused only on the hinging of the elbow and shoulder joints (see Figure 1 and Appendix A, Allen et al., 2017). Other studies, by contrast, have reported improvements on the 9HPT among patients with PD after they underwent exercises that were more physically demanding than that required by the Allen et al. (2017) exergame. Most notably is a study by Herz et al. (2013) that examined the effects of a Nintendo Wii-based rehabilitation on improving motor movement in patients with PD. In this study, patients played a sports-based exergame (Wii Sports) for 1 h three times a week for 4 weeks. The study concluded that patients demonstrated significant improvements on multiple motor measures, one being the 9HPT. We postulate that the more physically demanding and intensive nature (e.g., full-body exercise) of Wii Sports, compared to the Allen et al. (2017) exergame, provide the rationale behind the improvement of the 9HPT in Herz et al. (2013). This postulation is further supported by Duncan and Earhart (2011), who found that dancing, a fairly physically demanding intervention, led to significant improvement on the 9HPT among patients with PD.

## Forced exercise

Further support that physically demanding exercises may be necessary for motor improvement in PD comes from studies that have shown benefits of forcing participants to exercise at challenging levels. For example, Ridgel et al. (2009), and later Qutubuddin et al. (2013), observed significant motor improvements in patients who were forced to exercise at higher-than-preferred intensity levels on a stationary bicycle. By contrast, in those same studies, patients who exercised at a preferred intensity level did not show motor improvements. The benefits of forced exercise is further supported by Alberts et al. (2016), who reported the degree of motor improvements was equivalent between patients who underwent a forced exercise protocol (and were not on anti-PD medication) and those who were solely on anti-PD medication (and did not undergo the forced exercise protocol). Considering anti-PD medication is currently the standard form of care, such equivalence in motor improvement supports the possibility that physically-demanding exercises are necessary for motor improvement in PD. This perhaps explains the lack of observed motor improvement after patients had played the fairly sedentary exergame in Allen et al. (2017).

## Exercise and neurobiology

The relationship between exercise intensity and motor symptom alleviation is possibly explained by the impact of exercise on neurobiology. For example, in a study by Fontanesi et al. (2015), patients with PD who underwent multidisciplinary intensive rehabilitation treatment (featuring aerobic training) saw both improvements in motor function and increased brain-derived neurotrophic factor (BDNF)-tyrosine receptor kinase B (TrkB) pathway signaling. This relationship between exercise and increased BDNF expression has also been observed in other studies (e.g., Jeon and Ha, 2017; Park et al., 2017). Thus, physically demanding exercise protocols may induce improvement in motor function via an increased expression of BDNF, which has been implicated in synaptic transmission and formation, as well as long-term potentiation (Ohira and Hayashi, 2009).

## Conclusion

Allen et al. (2017) did not report any obvious benefits of their exergame in improving PD symptoms. This can potentially be attributed to the lack of an exercise-intensive protocol. If this were the case, high-intensity exergames can possibly be merged with virtual reality technology to create immersive experiences that effectively motivate participation while inducing greater symptom alleviation in patients with PD. Hence, future studies exploring such methodological adjustments in congruence with these developing technologies may better provide insight into exergames' viability as a rehabilitation protocol for PD.

## Author contributions

All authors listed have made a substantial, direct and intellectual contribution to the work, and approved it for publication.

### Conflict of interest statement

The authors declare that the research was conducted in the absence of any commercial or financial relationships that could be construed as a potential conflict of interest.
